# Cortical Structure in Relation to Empathy and Psychopathy in 800 Incarcerated Men

**DOI:** 10.1016/j.bpsgos.2026.100695

**Published:** 2026-01-23

**Authors:** Marcin A. Radecki, J. Michael Maurer, Keith A. Harenski, David D. Stephenson, Erika Sampaolo, Giada Lettieri, Giacomo Handjaras, Emiliano Ricciardi, Samantha N. Rodriguez, Craig S. Neumann, Carla L. Harenski, Sara Palumbo, Silvia Pellegrini, Jean Decety, Pietro Pietrini, Kent A. Kiehl, Luca Cecchetti

**Affiliations:** a Social and Affective Neuroscience Group, Molecular Mind Laboratory, IMT School for Advanced Studies Lucca, Lucca, Italy; b Autism Research Centre, Department of Psychiatry, University of Cambridge, Cambridge, United Kingdom; c The Mind Research Network, Albuquerque, New Mexico; d Molecular Mind Laboratory, IMT School for Advanced Studies Lucca, Lucca, Italy; e Department of Psychology, University of New Mexico, Albuquerque, New Mexico; f Department of Psychology, University of North Texas, Denton, Texas; g Department of Clinical and Experimental Medicine, University of Pisa, Pisa, Italy; h Department of Psychology, University of Chicago, Chicago, Illinois; i Department of Psychiatry and Behavioral Neuroscience, University of Chicago, Chicago, Illinois

**Keywords:** Antisocial behavior, Brain structure, Cortical gradients, Empathy, Multivariate prediction, Psychopathy

## Abstract

**Background:**

Reduced empathy is a hallmark of individuals with high (i.e., clinical) levels of psychopathy, who are overrepresented among incarcerated men. However, a comprehensive, well-powered mapping of cortical structure in relation to empathy and psychopathy is still lacking.

**Methods:**

In 804 incarcerated adult men, we administered the Perspective Taking (IRI-PT) and Empathic Concern (IRI-EC) subscales of the Interpersonal Reactivity Index (IRI), the Psychopathy Checklist-Revised (PCL-R; with interpersonal/affective [F1] and lifestyle/antisocial [F2] factors), and T1-weighted magnetic resonance imaging to quantify cortical thickness (CT), surface area (SA), and structural-covariance gradients.

**Results:**

PCL-R F1 was uniquely negatively related to IRI-EC, while PCL-R F2 was uniquely negatively related to IRI-PT. Cortical structure was not related to the IRI subscales. In contrast, CT was related to PCL-R F1 (mostly positively), SA was related to both PCL-R factors (only positively), and both cortical indices demonstrated out-of-sample predictive utility for PCL-R F1. Compared to men with low psychopathy, men with high psychopathy had uniquely lower IRI-EC scores and increased SA (but not CT); effect sizes across the cortex were largest in the paralimbic class and somatomotor network, while spatial overlap with meta-analytic task-based activations was highest for (social-)affective/sensory clusters. Finally, the total sample revealed anterior-posterior structural-covariance gradients; in men with high psychopathy, the gradient of CT (but not SA) was globally compressed.

**Conclusions:**

Men with high psychopathy had reduced empathic concern, increased SA, and a compressed macroscale organization of CT, indicating selective co-alterations in empathy and cortical structure. Future work should build on these novel insights in both the general and incarcerated populations to inform the treatment of psychopathy.

## Plain Language Summary

Reduced empathy is a hallmark of individuals with high (i.e., clinical) levels of psychopathy. These individuals are overrepresented among incarcerated men and are at high risk for future antisocial behavior. However, relationships of cortical structure with empathy and psychopathy remain poorly understood. Here, we measured empathy and psychopathy alongside cortical thickness (CT) and surface area (SA) in ∼800 incarcerated men. Men with high psychopathy had reduced empathic concern, increased SA, and a distinct pattern of the way CT varied across the cortex. These novel insights into behavior and brain structure in a large antisocial sample aim to inform the treatment of psychopathy.

Empathy comprises desirable psychological traits that allow us to understand another person’s mental states (cognitive empathy), share their affective states (affective empathy), and feel concern for their well-being (empathic concern) (1, 2, 3, 4, 5, 6). Reduced empathy, particularly affective empathy and empathic concern, is a hallmark of individuals with high (i.e., clinical) levels of psychopathy (7, 8, 9) [for meta-analyses, see (10,11)]. More broadly, psychopathy is a constellation of interpersonal/affective traits (e.g., reduced empathy) and lifestyle/antisocial traits (e.g., impulsivity), as operationalized by factors 1 and 2 of the Psychopathy Checklist-Revised (PCL-R), respectively (12, 13, 14). The prevalence of high psychopathy based on the PCL-R approximates 1.2% in the general adult population; it is higher among incarcerated individuals (up to 25%) and among males/men than females/women (up to 3-fold) (15). Because psychopathy incurs societal costs that reach hundreds of billions of USD per annum through violence, crime, and recidivism (16), we need to advance its psychological and neuroscientific characterization to improve long-term treatment outcomes (17, 18, 19).

It remains unknown how brain structure is related to empathy [e.g., as measured by the Interpersonal Reactivity Index (IRI) (20, 21, 22)] in the incarcerated population. Even in the general population, no structural meta-analysis has yet been conducted, further motivating investigation into the underlying brain structure [for study examples, see (23, 24, 25)]. Functional meta-analyses highlight default mode hubs for cognitive empathy (e.g., medial prefrontal cortex), ventral attention hubs for affective empathy (e.g., insula), and reward-related regions for empathic concern (e.g., ventromedial prefrontal cortex and striatum) (26, 27, 28, 29, 30, 31). Altered function of these and more distributed regions, particularly during social and affective processing (32, 33, 34, 35, 36, 37, 38, 39, 40), provides insights into reduced empathy and other psychopathic traits [for meta-analyses, see (41, 42, 43)]. While a structural literature on psychopathy exists [e.g., (44, 45, 46, 47, 48, 49)], it suffers from limited replicability, in part owing to small sample sizes (50), and is almost exclusively focused on voxel-based gray matter volume (GMV), with the most common result being reduced GMV [for a meta-analysis, see (51); also see (52)]. Surprisingly, no study on psychopathy reported to date has investigated (raw) cortical surface area (SA), let alone alongside cortical thickness (CT), 2 critical constituents of cortical GMV [but see (53)]. Given that CT and SA diverge in their evolutionary (54), genetic (55), developmental (56), and psychiatric (57) profiles, it is crucial to fractionate them, as they may differentially map onto multidimensional empathy and psychopathy. Indeed, SA has already demonstrated a higher sensitivity than CT to broadly construed antisocial behavior [(58,59); also see (60)]. To further enhance the generalizability of such brain-behavior relationships, a multivariate attempt at out-of-sample prediction is recommended (61, 62, 63), especially since CT and SA have not yet demonstrated predictive utility for psychopathy [but see (64)].

To overcome some of the limitations of investigating raw cortical structure, a novel organizational framework is offered by gradients—topographical patterns of regional similarity, including structural covariance (65, 66, 67), that are embedded in a low-dimensional space (68, 69, 70). Based on meta-analytic data, CT gradients have been shown to differ across major psychiatric conditions in a transdiagnostic fashion (71, 72, 73) that dovetails with differences along the primary (i.e., unimodal-transmodal) axis of intrinsic connectivity in schizophrenia (74), autism (75), or depression (76). In these conditions, the unimodal-transmodal gradient has been observed to be compressed [as opposed to expanded (77)]. Such compression corresponds to a smaller gradient range and indicates reduced differentiation between its ends, where the opposing unimodal/sensorimotor regions and transmodal/association regions have more similar connectivity patterns (78). Importantly, an anterior-posterior compression has also been observed for CT in schizophrenia (79). It remains to be established whether individuals with high psychopathy may exhibit similar differences in the macroscale organization of either CT or SA.

In sum, a comprehensive, well-powered mapping of cortical structure in relation to empathy and psychopathy is still lacking. There is thus a need to investigate CT alongside SA in an interpretable way that is facilitated by theories of psychopathy [e.g., regarding laminar differentiation (80, 81, 82)] and brain function [e.g., regarding intrinsic connectivity (83)]. Simultaneously, this gap presents an opportunity to clarify statistically unique relationships between the IRI and PCL-R, 2 of the most widely used measures of empathy and psychopathy, respectively. Here, we analyze data from a large sample of incarcerated adult men (*N* = 804), grounding our expectations in meta-analyses documenting negative relationships of psychopathy with both empathy (10,11) and cortical GMV (51). We asked the following 5 overarching questions (also see Statistical Analysis):

Q1: How is psychopathy related to empathy given the multidimensionality of both constructs and their potential for unique relationships?

Q2: How is cortical structure, distinguishing CT and SA, related to empathy and psychopathy?

Q3: Can cortical structure predict empathy and psychopathy in out-of-sample individuals?

Q4: How does cortical structure differ among individuals with high (i.e., clinical) levels of psychopathy?

Q5: How do structural-covariance gradients differ among these individuals?

## Methods and Materials

### Participants

Nine hundred twelve adult men (gender self-reported) were recruited by the Mind Research Network (MRN) from correctional facilities in the southwestern and midwestern United States and had partial data available, including a T1-weighted magnetic resonance imaging (MRI) scan. We included 804 men who sequentially met the following criteria: 1) passed structural-data quality control (*n*_excluded_ = 105; see MRI in the Supplement); 2) had data on empathy, psychopathy, age, and IQ (*n*_excluded_ = 1); and 3) had an IQ ≥ 70 (*n*_excluded_ = 2) (for participant characteristics, see Table 1). Among the included participants, 723 (∼90%) reported having committed a violent crime (e.g., murder), 715 (∼89%) a nonviolent crime (e.g., theft), and 634 (∼79%) both a violent and nonviolent crime [based on a classification similar to (84); see Covariates in the Supplement]. All participants provided written informed consent, and all research procedures were approved by the Institutional Review Board of the University of New Mexico or the Ethical and Independent Review Services for data collection post June 2015.

**Table 1:** Participant Characteristics

	Total, *N* = 804	Low Psychopathy, *n* = 289	High Psychopathy, *n* = 178	Cohen’s *d*, *p*
Age, Years	33.78 ± 8.23 [18.75–62.83]	34.20 ± 8.51 [18.75–60.56]	33.69 ± 8.19 [19.47–62.83]	−0.06, .521
IQ	97.88 ± 13.14 [71–137]	98.56 ± 13.26 [72–134]	100.03 ± 12.68 [72–137]	0.11, .277
PCL-R	22.85 ± 7.06 [3.20–38]	15.15 ± 3.83 [3.20–20]	32.04 ± 1.96 [30–38]	5.19, 7 × 10^−74^∗
PCL-R F1	7.90 ± 3.61 [0–16]	4.74 ± 2.58 [0–12]	12.14 ± 1.86 [8–16]	3.17, 2 × 10^−70^∗
PCL-R F2	12.79 ± 4.01 [1.10–20]	8.97 ± 3.26 [1.10–17]	16.85 ± 1.89 [11–20]	2.80, 5 × 10^−68^∗
Race, White	534	225	100	3 × 10^−7^∗
SU	21.63 ± 21 [0–158]	19.44 ± 19.81 [0–107]	22.34 ± 19.44 [0–111]	0.15, .022∗
Adj. SU	7.01 ± 3.64 [0–18.89]	6.40 ± 3.74 [0–15.19]	7.43 ± 3.55 [0–18.89]	0.28, .008∗
TIV, mm^3^	1.58 × 10^6^ ± 1.5 × 10^5^ [9.6 × 10^5^–2.0 × 10^6^]	1.60 × 10^6^ ± 1.4 × 10^5^ [1.1 × 10^6^–2.0 × 10^6^]	1.58 × 10^6^ ± 1.6 × 10^5^ [1.1 × 10^6^–1.9 × 10^6^]	−0.13, .293
Euler No.	11.88 ± 4.95 [0–24]	12.20 ± 4.99 [3–24]	12.31 ± 5 [3–23]	0.02, .753

Values are presented as mean ± SD [range] (or *n* for race) as well as Cohen’s *d*s for men with high psychopathy (PCL-R ≥ 30) vs. men with low psychopathy (PCL-R ≤ 20), with *p* values derived from Wilcoxon’s rank-sum test (or Pearson’s χ^2^ test for race). IQ is the full-scale IQ estimate based on the Vocabulary and Matrix Reasoning subtests of the Wechsler Adult Intelligence Scale-III or Wechsler Abbreviated Scale of Intelligence-II. PCL-R F1 is the interpersonal/affective factor. PCL-R F2 is the lifestyle/antisocial factor (*n* = 778). Race is White (vs. non-White, *n* = 789). SU is total years of substance use based on the Addiction Severity Index, 5th ed. (*n* = 748). Adj. SU is age corrected and square root–transformed (to correct for opportunity to use and skewness) total years of substance use (*n* = 748). Euler No. is the total number of topological defects in the cortical surface prior to fixing in the FreeSurfer pipeline (to be treated as a measure of structural-data quality).

∗*p* < .05, uncorrected.

PCL-R, Psychopathy Checklist-Revised; SU, substance use; TIV, total intracranial volume.

We also included the male sample from the Human Connectome Project (HCP) Young Adult S1200 release (85, 86, 87) with structural MRI and IQ data (*N* = 501) (Table S1). This dataset has been used to derive the canonical anterior-posterior gradient of CT (67), which then served as an external reference in the aforementioned work on psychiatric differences (71, 79). Given that structural-covariance gradients in our total sample served as the alignment reference for psychopathy groups (Q5), it was important to evaluate their consistency with normative patterns (i.e., those in the general male population, represented by the HCP sample) and to conduct a related sensitivity analysis by psychopathy group. Note that the HCP did not include our measures of empathy and psychopathy.

### Magnetic Resonance Imaging

On the grounds of the correctional facilities, high-resolution T1-weighted MRI scans were acquired with the MRN’s mobile scanner (i.e., 1.5T Siemens MAGNETOM Avanto with a 12-channel, multi-echo magnetization-prepared rapid acquisition gradient-echo [MPRAGE] pulse sequence). The scanning parameters were as follows: TR = 2530 ms; TE = 1.64, 3.50, 5.36, and 7.22 ms; inversion time = 1100 ms; flip angle = 7°; slice thickness = 1.3 mm; matrix size = 256 × 256, yielding 128 sagittal slices with an in-plane resolution of 1.0 × 1.0 mm.

Each scan underwent the standard recon-all pipeline in FreeSurfer version 7.4.1 [https://surfer.nmr.mgh.harvard.edu/ (88)] and was parcellated in the HCP-MMP1.0 atlas to delineate 360 regions (89). For quality control and HCP data, see MRI in the Supplement.

### Empathy

Empathy was measured with the Perspective Taking (IRI-PT) and Empathic Concern (IRI-EC) subscales of the IRI (21), a self-report questionnaire of trait empathy widely used in both nonincarcerated and incarcerated samples. Across these samples, IRI-PT is traditionally labeled a measure of cognitive empathy and IRI-EC a measure of affective empathy (10,11,90, 91, 92, 93). We agree with the labeling of IRI-PT, but the labeling of IRI-EC conflates affective empathy with empathic concern; accordingly, we label IRI-EC a measure of the latter. More specifically, IRI-PT assesses the “tendency to spontaneously adopt the psychological point of view of others,” while IRI-EC assesses the “feelings of sympathy and concern for unfortunate others” [(21), pp. 113–114]. Each subscale includes 7 items scored on a 5-point Likert scale ranging from “does not describe me well” (0 points) to “describes me very well” (4 points) (for all items, see Table S2). Thus, possible scores range from 0 to 28 points per subscale, with higher scores indicating higher levels of empathy. The remaining Fantasy and Personal Distress subscales of the IRI were not included, as they are less frequently used to distinguish cognitive empathy from affective empathy and empathic concern (92) and are less related to psychopathy (10) (also see Internal consistency in the Supplement).

### Psychopathy

Psychopathy was measured with Hare’s PCL-R (13). All PCL-R scores were based on both a semistructured interview and institutional-file review conducted by the MRN’s research staff with a bachelor’s degree or higher following rigorous training designed and supervised by KAK. The MRN has historically completed independent double ratings on ∼10% of all PCL-R interviews, obtaining excellent rater agreement (45). The PCL-R includes 20 items that largely correspond to 2 factors, interpersonal/affective (F1; 8 items) and lifestyle/antisocial (F2; 10 items) (for all items, see Table S3). Each item is scored 0, 1, or 2 points, indicating no evidence, some evidence, and pervasive evidence, respectively. The total score is a sum across the 20 items, thus ranging from 0 to 40 points, with higher scores indicating higher levels of psychopathy. PCL-R total and PCL-R F1 scores were available for the total sample (i.e., *N* = 804, where *n* = 582 and *n* = 798 had complete item-level data, respectively); PCL-R F2 score was available for *n* = 778 (where *n* = 617 had complete item-level data). For items omitted due to insufficient information, we used a prorating formula to estimate the total and factor scores with possible decimals. Also see Internal consistency in the Supplement.

Following both the PCL-R guideline (13) and extensive work with incarcerated adult males/men [e.g., (32, 33, 34, 35, 36,40,94, 95, 96)], we defined “high” psychopathy as a PCL-R score of ≥30 and “low” psychopathy as a PCL-R score of ≤20. Beyond comparability with the literature, this extreme-groups approach allowed us to specifically investigate participants with clinical levels of psychopathy against a clearly isolated nonclinical group, knowing that those highest in psychopathy are at the highest risk for future antisocial behavior (97, 98, 99). Furthermore, this approach allowed for comparability with inherently categorical analyses (Q5) while not necessarily reducing statistical power (100). Therefore, we focus on these categorical analyses below (alongside analyses for PCL-R F1 and PCL-R F2), but for completeness, we report analyses for the PCL-R total score (Q1–Q3) in the Supplement.

### Statistical Analysis

Statistical analyses were conducted in MATLAB version R2020b (The MathWorks, Inc.; https://www.mathworks.com/) and are described in more detail in the Supplement (alongside Mesulam’s classes and Yeo’s networks; Multivariate prediction; Meta-analytic task-based activations; and Structural-covariance gradients). Briefly, for Q1, we tested for relationships of psychopathy (PCL-R F1 and PCL-R F2; PCL-R total score in the Supplement) with empathy (IRI-PT and IRI-EC), controlling for age and IQ in a robust linear regression. We then tested empathy by psychopathy group (high vs. low), controlling for the same covariates. For Q2, we tested for relationships of cortical structure (CT and SA) with empathy and psychopathy at a false discovery rate (FDR) of *p* < .05 (101), controlling for age and IQ with CT and additionally for total intracranial volume with SA. For interpretability, we compared effect sizes across the cortex by 4 Mesulam’s classes (82) and 7 Yeo’s networks (83) using Wilcoxon’s rank-sum test. For Q3, which complemented the univariate analyses for Q2, we took a multivariate approach with a train-test split and cross-validation to predict empathy and psychopathy from cortical structure (corrected for the same covariates) using ridge regression. For Q4, we tested for global and regional differences in cortical structure by psychopathy group, controlling for the same covariates. We then compared effect sizes across the cortex as above; for additional psychological interpretability, we computed spatial overlap between results at *p*_FDR_ < .05 and meta-analytic task-based activations from Schurz *et al.* (31) and Neurosynth (102). Finally, for Q5, we tested for psychopathy-group differences in structural-covariance gradients using Kolmogorov-Smirnov’s test (globally) and Wilcoxon’s signed-rank test (at the class/network level). Here, gradient consistency between the total and HCP samples was evaluated through spatial correlation (103,104). Across the analyses, Bonferroni’s correction was applied where appropriate.

## Results

### Empathy and Psychopathy (Q1)

**Figure 1:**
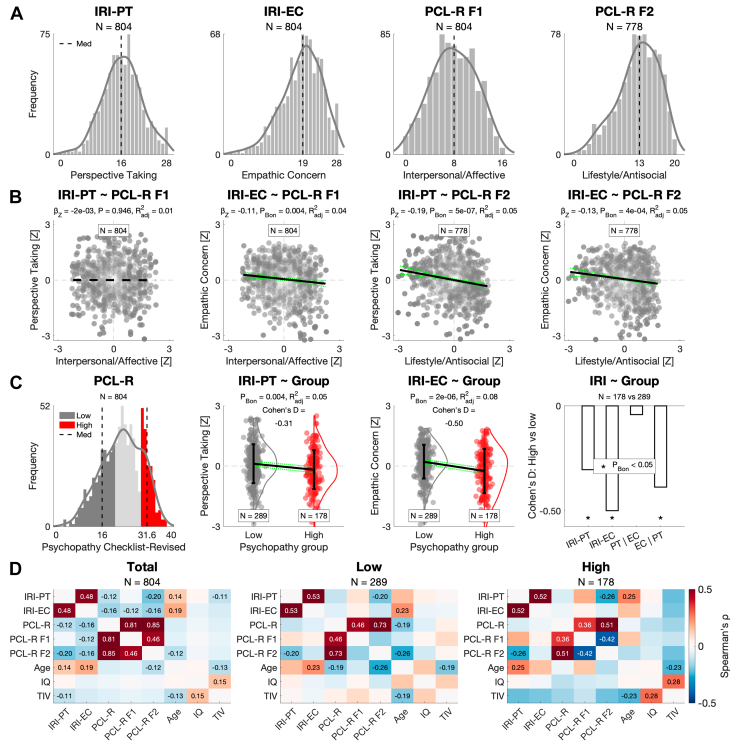
Psychopathy in relation to empathy (question 1 [Q1]). **(A)** Distribution of scores in the total sample for the Interpersonal Reactivity Index Perspective Taking subscale (IRI-PT), IRI Empathic Concern subscale (IRI-EC), Psychopathy Checklist-Revised factor 1 (PCL-R F1), and PCL-R factor 2 (PCL-R F2). **(B)** Relationships of PCL-R F1 and PCL-R F2 (negative, if any) with IRI-PT and IRI-EC, controlling for age and IQ in a robust linear regression with Bonferroni’s correction across the IRI subscales. **(C)** From left to right: distribution of the PCL-R total score, depicting men with low psychopathy (PCL-R ≤ 20; dark gray) and men with high psychopathy (PCL-R ≥ 30; red); lower scores on IRI-PT and IRI-EC in men with high psychopathy, controlling for age and IQ with Bonferroni’s correction across the IRI subscales; lower score in men with high psychopathy on IRI-EC but no longer IRI-PT when additionally controlling for the other IRI subscale. **(D)** Sample-specific Spearman’s correlation matrices, with numeric effect sizes displayed at *p*_Bon_ < .05 following correction across the 28 tests. TIV, total intracranial volume.

In the total sample, we first tested for relationships of psychopathy with empathy (Q1). PCL-R F1 had a negative relationship with IRI-EC, while PCL-R F2 had a negative relationship with both IRI subscales (Figure 1A, B, Figure S1, and Table S4). In categorical analyses, men with high psychopathy scored lower on both IRI subscales, with a larger effect size for IRI-EC. These relationships became clearer when additionally controlling for the other IRI subscale; PCL-R F1 was uniquely negatively related to IRI-EC, while PCL-R F2 was uniquely negatively related to IRI-PT. Furthermore, the group difference on IRI-PT became insignificant, while the group difference on IRI-EC remained significant, and this did not change when additionally controlling for race and substance use (Figure 1C and Table S5). For sample-specific correlations, see Figure 1D.

### Cortical Structure, Empathy, and Psychopathy (Q2–Q3)

**Figure 2:**
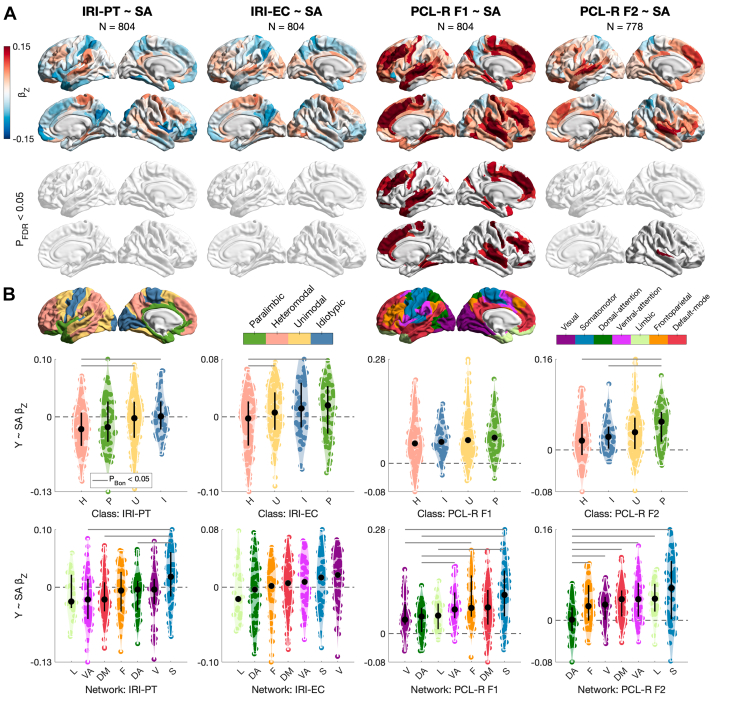
Cortical surface area (SA) in relation to empathy and psychopathy (question 2 [Q2]). **(A)** Relationships of SA (positive, if any) with the Interpersonal Reactivity Index Perspective Taking subscale (IRI-PT), IRI Empathic Concern subscale (IRI-EC), Psychopathy Checklist-Revised factor 1 (PCL-R F1), and PCL-R factor 2 (PCL-R F2), controlling for age, IQ, and total intracranial volume in a robust linear regression with a false discovery rate (FDR) correction. Grayed-out parcels are insignificant at *p*_FDR_ < .05. **(B)** Standardized betas across the cortex by Mesulam’s class and Yeo’s network, median-ordered, and tested for distribution differences using Wilcoxon’s rank-sum test with Bonferroni’s correction within class (6 comparisons) or network (21 comparisons).

Next, we tested for relationships of CT and SA with empathy and psychopathy (Q2). CT was not related to the IRI subscales or PCL-R F2. However, CT in 16 parcels had a positive relationship with PCL-R F1, while CT in 6 parcels had a negative relationship. Across the cortex, effect sizes for PCL-R F1 were largest in the heteromodal class of Mesulam and the frontoparietal network of Yeo (positive median in both cases), with differentiation by both class (e.g., heteromodal > paralimbic) and network (e.g., frontoparietal > visual) (Figures S2 and S3; Table S6). Similarly to CT, SA was not related to the IRI subscales. In contrast, SA had a positive relationship with PCL-R F1 in 103 parcels and with PCL-R F2 in the same 3 parcels in the right superior-temporal/auditory cortex. For both PCL-R factors, effect sizes were largest in the paralimbic class and somatomotor network (positive median in both cases), with differentiation by class for PCL-R F2 (e.g., paralimbic > heteromodal) and by network for both PCL-R factors (e.g., somatomotor > dorsal attention) (Figure 2 and Tables S7–S9). In sensitivity analyses, the null CT and SA results for the IRI subscales did not change when leveraging their psychometrically modified versions (see Internal consistency in the Supplement; Figure S4), while the positive CT and SA results for PCL-R F1 (and less so for PCL-R F2) remained highly consistent when taking 2 alternative approaches to structural-data quality control (see MRI in the Supplement; Figure S5).

**Figure 3:**
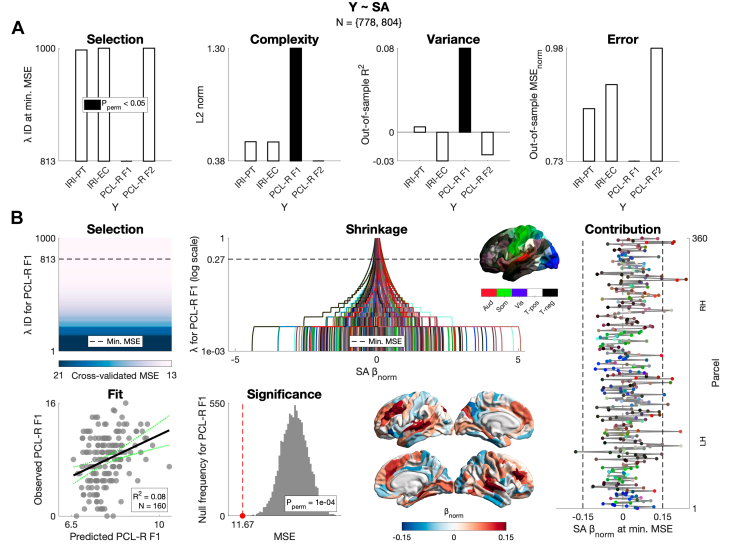
Multivariate prediction of empathy and psychopathy from cortical surface area (SA) (question 3 [Q3]). **(A)** For the Interpersonal Reactivity Index Perspective Taking subscale (IRI-PT), IRI Empathic Concern subscale (IRI-EC), Psychopathy Checklist-Revised factor 1 (PCL-R F1), and PCL-R factor 2 (PCL-R F2), we inform on: model selection using cross-validated ridge regression (i.e., lambda corresponding to the minimum cross-validated mean squared error [MSE] at which the model was selected); model complexity (i.e., Euclidean norm of the final beta vector); variance explained (i.e., out-of-sample coefficient of determination); and prediction error (i.e., out-of-sample MSE divided by the maximum possible score and thus normalized). SA was corrected for age, IQ, and total intracranial volume separately in the training (*n* = 644/623) and test (*n* = 160/155) sets. Among the 4 variables, only PCL-R F1 was able to be predicted (*R*^2^ = 0.08 [95% CI, 0.02–0.13], *p*_perm_ = 1 × 10^−4^). **(B)** For PCL-R F1, we inform on model selection, beta shrinkage, final beta vector, predicted-observed fit, and significance based on permutation for out-of-sample MSE (*N*_perm_ = 10,000). LH, left hemisphere; RH, right hemisphere.

We then tested for multivariate, predictive relationships of CT and SA with empathy and psychopathy using a train-test split and cross-validation (Q3). Still, CT or SA were not related to the IRI subscales. In contrast, the univariate relationships of both CT and SA with PCL-R F1 (but not PCL-R F2) were corroborated; CT explained ∼6% of the out-of-sample variance in PCL-R F1, while SA explained ∼8% (Figure 3 and Figure S6).

### Cortical Structure by Psychopathy Group (Q4)

**Figure 4:**
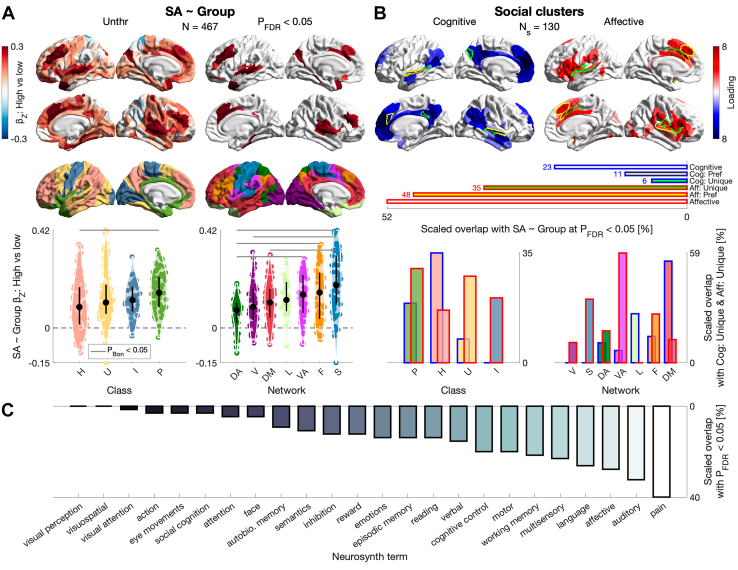
Cortical surface area (SA) by psychopathy group (question 4 [Q4]). **(A)** Differences in SA by psychopathy group (high [*n* = 178] vs. low [*n* = 289]), controlling for age, IQ, and total intracranial volume in a robust linear regression with a false discovery rate (FDR) correction; 65 parcels showed an increase in men with high psychopathy. Across the cortex, standardized betas were median-ordered by class/network and tested for distribution differences using Wilcoxon’s rank-sum test with Bonferroni’s correction within class (6 comparisons) or network (21 comparisons). Total SA was increased in men with high psychopathy as well, controlling for the same covariates (β_Z_ = 0.25 [95% CI, 0.14−0.36], *p* = 9 × 10^−6^, adj. *R*^2^ = 0.67, Cohen’s *d* = 0.39). **(B)** Meta-analytic clusters of social-cognitive and social-affective processing across 130 studies (31). The FDR-corrected cluster of SA increases overlapped multiple times more with social-affective than social-cognitive clusters across different social-cluster thresholds. “Cognitive” and “Affective” are baseline clusters; “Cog: Pref” and “Aff: Pref” are preferential clusters (baseline cluster 1 > baseline cluster 2; overlap delineated in yellow [for parcels shared between the baseline clusters] and green [for unique parcels]); “Cog: Unique” and “Aff: Unique” are unique clusters (baseline cluster 1 > 0 and baseline cluster 2 = 0; green). Below, social-cluster overlap with the classes/networks (for the unique clusters, which showed the largest proportional difference). **(C)** Overlap between the FDR-corrected cluster and Neurosynth clusters (102). Class: P, paralimbic; H, heteromodal; U, unimodal; I, idiotypic. Network: V, visual; S, somatomotor; DA, dorsal attention; VA, ventral attention; L, limbic; F, frontoparietal; DM, default mode. Unthr, unthresholded.

Next, we tested for global and regional differences in cortical structure by psychopathy group (Q4). There was no difference in CT (Figure S7). In contrast, men with high psychopathy had increased total SA. Regionally, there was an increase in 65 parcels (Figure 4A and Table S10); additionally controlling for race and substance use yielded highly similar results (Figure S8). More specifically, effect sizes across the cortex were largest in the paralimbic class and somatomotor network (positive median in both cases), with differentiation by both class (i.e., paralimbic > heteromodal) and network (e.g., somatomotor > dorsal attention). We then computed spatial overlap between the FDR-corrected cluster of SA increases and meta-analytic brain-behavior data. First, using task-based activations underlying social-cognitive and social-affective processing (Figure S9), the SA increases overlapped multiple times more with social-affective than social-cognitive clusters (Figure 4B). Secondly, using task-based activations across 24 wide-ranging terms from Neurosynth (Figure S10), the overlap was highest for affective/sensory terms (top 2: “pain,” “auditory”) and lowest for visual terms (bottom 2: “visual perception,” “visuospatial”) (Figure 4C).

### Structural-Covariance Gradients by Psychopathy Group (Q5)

**Figure 5:**
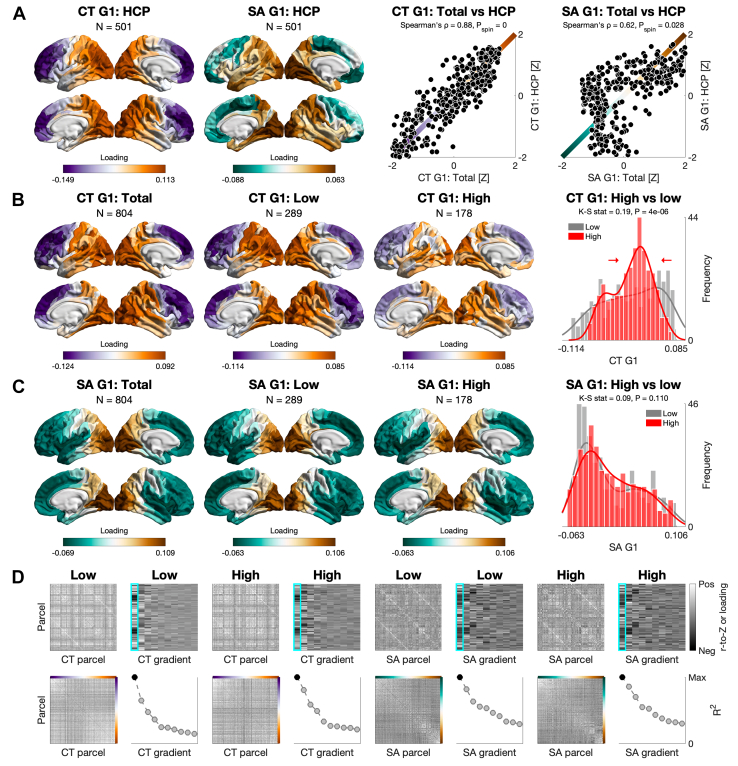
Macroscale organization of cortical thickness (CT) and surface area (SA) by psychopathy group: Global analysis (question 5 [Q5]). **(A)** Primary gradients of CT and SA in the Human Connectome Project (HCP) sample (*N* = 501) and their spatial correlations with those in the total sample (*N* = 804) following spin permutation (*N*_perm_ = 1000). In both datasets, CT was corrected for age and IQ, while SA was additionally corrected for total intracranial volume. **(B)** Primary gradients of CT in the total, low-psychopathy, and high-psychopathy samples. The CT gradient was compressed in men with high psychopathy compared to men with low psychopathy using Kolmogorov-Smirnov’s test. **(C)** Primary gradients of SA in the 3 samples. The SA gradient did not differ by psychopathy group using the same test. **(D)** The 2-by-2 left-hand tiles: sample-specific structural-covariance matrix (top left), array of the first 10 gradients (top right), structural-covariance matrix ordered by the primary gradient (bottom left), and the first 10 gradients ordered by the proportion of variance explained (i.e., scaled eigenvalues; bottom right). For visualization, all matrices were set to the range [−0.5 to 0.5]; all arrays were set to the minimum–maximum range.

**Figure 6:**
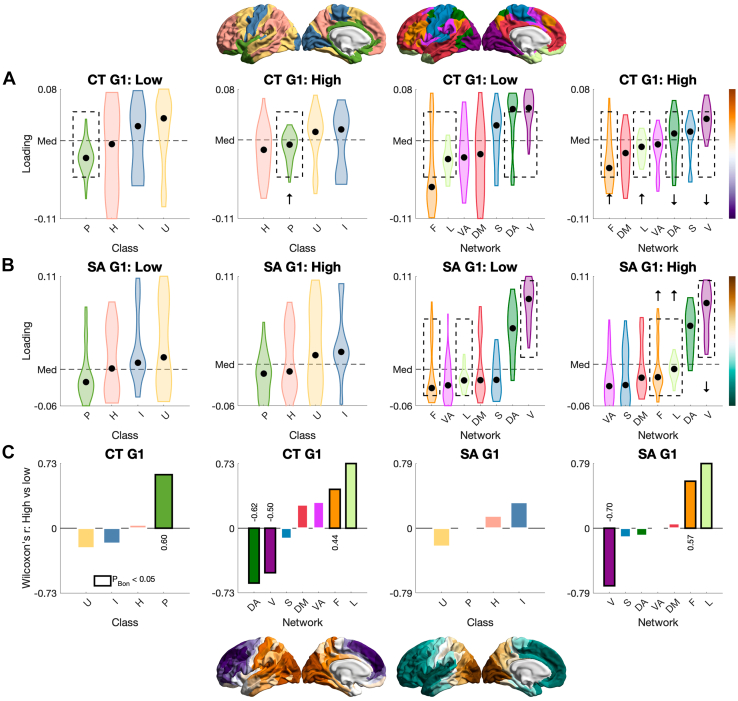
Macroscale organization of cortical thickness (CT) and surface area (SA) by psychopathy group: Local analysis (question 5 [Q5]). **(A)** CT and **(B)** SA gradients by class/network in men with low psychopathy (*n* = 289) and men with high psychopathy (*n* = 178), median-ordered. Both gradients in men with low psychopathy, and the CT gradient in men with high psychopathy, traversed a frontoparietal-to-visual axis; the SA gradient in men with high psychopathy traversed a ventral attention–to-visual axis instead. **(C)** Using Wilcoxon’s signed-rank test with Bonferroni’s correction within class (4 comparisons) or network (7 comparisons), both gradients in men with high psychopathy differed in a compression-oriented manner—where the class/network median was pulled toward the center (i.e., median across the classes/networks)—in the visual, limbic, and frontoparietal networks. The CT gradient further differed in this manner in the paralimbic class and dorsal attention network. Class: P, paralimbic; H, heteromodal; U, unimodal; I, idiotypic. Network: V, visual; S, somatomotor; DA, dorsal attention; VA, ventral attention; L, limbic; F, frontoparietal; DM, default mode.

Finally, we tested structural-covariance gradients by psychopathy group (Q5). First, the total sample revealed primary gradients of CT and SA that traversed anterior-posterior axes and were spatially correlated with those in the HCP sample (Figure 5A–C and Figure S11). We then tested for psychopathy-group differences in the gradients aligned to those in the total sample to ensure that they traversed the same axes and were directly comparable (Figure 5D; for raw gradients, see Figure S12). The primary gradient of CT was compressed in men with high psychopathy, such that its distribution had a smaller range and was pulled toward the center. Such compression was not observed for SA. In sensitivity analyses for CT, compression was also observed for high versus moderate psychopathy (to a smaller extent); when lowering the high-psychopathy threshold; when matching the high- and low-psychopathy groups for size; and when using different alignment templates, including from the HCP sample (Figure S13). Furthermore, aggregating gradient loadings by class/network yielded compression-oriented differences in the visual, limbic, and frontoparietal networks for both cortical indices, and further differences in the paralimbic class and dorsal attention network for CT (Figure 6).

## Discussion

A comprehensive, well-powered mapping of cortical structure in relation to empathy and psychopathy has been lacking. We addressed this gap in ∼800 incarcerated men through 5 overarching questions.

As expected, psychopathy had negative relationships with empathy (Q1). PCL-R F1 had a negative relationship with IRI-EC but not IRI-PT, while PCL-R F2 had a negative relationship with both IRI subscales. Controlling for the other subscale revealed statistically unique contributions of PCL-R F1 to IRI-EC and of PCL-R F2 to IRI-PT. In categorical analyses for high (i.e., clinical) levels of psychopathy, men with high psychopathy scored lower on both IRI subscales, but only the difference on IRI-EC (which was larger) proved unique. Evoking a mirror-opposite image with autism (7,105, 106, 107, 108, 109), this is consistent with meta-analytic evidence that psychopathy is primarily associated with reduced affective empathy and empathic concern rather than cognitive empathy (10,11) [for a similar conclusion based on self-report data, see, e.g., (110)]. We further add to this literature by revealing a pattern of unique relationships, suggesting that the psychopathic reduction in cognitive empathy—meta-analytically replicable also based on performance data (111, 112, 113)–may depend on the reduction in empathic concern, at least in part.

SA had positive relationships with psychopathy (Q2). This was observed for 103 of 360 parcels for PCL-R F1 and 3 parcels for PCL-R F2. The superior-temporal/auditory cortex, playing a role in affective-speech processing (114), emerged for both PCL-R factors, with effect sizes across the cortex being largest in the paralimbic class and somatomotor network. These SA increases were in contrast to what we expected based on meta-analytic GMV reductions observed for male psychopathy (51), knowing that cortical GMV closely tracks SA genetically and phenotypically (115). However, it is important to note substantial differences of this meta-analysis; it synthesized voxel-based morphometry studies across mixed (i.e., nonincarcerated and incarcerated) samples ranging from as few as *N* = 12 to *N* = 254 (total *N* = 519). In contrast to the PCL-R factors, we did not observe any relationship of SA with the IRI subscales, which raises questions about the latter’s reliability (116) and calls for measuring empathy beyond self-report in forensic neuroimaging. Regarding CT, we observed more circumscribed relationships with PCL-R F1 compared to SA (mostly positive) but not with any other behavioral variable tested. Together with our multivariate prediction of PCL-R F1 (but not PCL-R F2), where SA explained more out-of-sample variance than CT did (Q3), our results suggest that PCL-R F1 is more neuroanatomically distinctive than PCL-R F2, and that this is better captured by SA than CT. Importantly, to our knowledge, this is the first evidence of any relationship between SA and psychopathy.

Corroborating the dimensional results, men with high psychopathy had increased SA (Q4). These increases spanned 65 parcels and also covered the superior-temporal/auditory cortex; additional parcels covered the anterior cingulate, insula, temporal pole, or dorsolateral, dorsomedial, and orbital prefrontal cortices. Again, effect sizes across the cortex were largest in the paralimbic class and somatomotor network. The paralimbic class has been hypothesized to be especially relevant to psychopathy under the paralimbic-dysfunction model (81). While the SA increases we observed indeed highlight paralimbic relevance, they do not readily align with the GMV reductions posited by the model and commonly reported (17,51,80). In our study, what further highlights the relevance of (social-)affective/sensory regions to psychopathy is the spatial overlap of the SA increases with meta-analytic task-based activations. In particular, the SA increases overlapped multiple times more with clusters of social-affective than social-cognitive processing across 130 studies (31), while across 24 wide-ranging brain-behavior meta-analyses (102), the overlap was highest for affective/sensory terms (“pain” and “auditory”). In contrast, we observed no psychopathy-group difference in CT. While these results are consistent with a higher sensitivity of SA than CT to broadly construed antisocial behavior, they are again inconsistent with the effect direction. This is because reductions rather than increases in SA have been reported among antisocial individuals, although drawn largely (59) or even exclusively (58) from the general rather than incarcerated population. Therefore, it will be essential for future work to reconcile the SA increases we observed for psychopathy, dimensionally and categorically, with both the GMV reductions (meta-analytically) observed for psychopathy and the SA reductions observed for broader antisocial behavior [but see (50,80) for systematic reviews noting GMV increases for psychopathy]. To enhance the developmental framing of the SA increases observed here, the cellular and physical mechanisms driving cortical expansion and folding—including neural-progenitor proliferation, tangential neuronal migration, or growth-induced mechanical stress—should be considered (117, 118, 119).

Finally, men with high psychopathy had a compressed macroscale organization of CT (Q5). The primary gradient in the total sample traversed an anterior-posterior axis for CT [as reported across the sexes in the HCP, likely mirroring the temporal sequence of neurogenesis (67)] and a similar axis for SA [as reported in the genomic literature (120)]. Both gradients were spatially correlated with those in the male HCP sample, suggesting their consistency with normative patterns, albeit more evidently for CT than SA. When testing for psychopathy-group differences along these axes, we observed a globally compressed gradient of CT but not SA in men with high psychopathy. At the class/network level, this group further showed compression-oriented differences for both gradients, with converging and largest differences in the limbic network. CT gradients are known to differ across (71, 72, 73) and within (79) major psychiatric conditions, as is the primary functional gradient in terms of compression (74, 75, 76). We provide the first evidence that individuals with high psychopathy may exhibit similar macroscale properties of the cortex, with reduced differentiation between anterior/transmodal regions (e.g., frontoparietal) and posterior/unimodal regions (e.g., visual)—which anchored the ends of our CT gradients. It is an open question whether such reduced differentiation reflects disrupted integration and segregation from a connectomic perspective (78,121,122), which sets the stage for probing psychopathy-related differences along the unimodal-transmodal axis itself (70). This axis not only recapitulates the anterior-posterior axis of CT (67) but may be clinically compressed along with the CT axis, as suggested for schizophrenia (79).

There are limitations to our study. For example, a performance test of empathy may have yielded additional insights. Indeed, our use of the IRI cannot be conclusive given the questionable correspondence between self-reported and tested empathy (123), and the potential for social-desirability bias and metacognitive deficits in the incarcerated population. Importantly, our results may not generalize to nonincarcerated samples (124) or to female samples, given sex/gender differences in empathy (125, 126, 127) and cortical structure (128), beyond sex/gender differences in psychopathy (15). Thus, recruiting from the general and incarcerated female populations should be prioritized. Furthermore, the roles of other macrostructural indices (e.g., gyrification, sulcal depth, curvature), microstructural indices (e.g., diffusion-based), and subcortical indices remain to be elucidated.

### Conclusions

In conclusion, men with high psychopathy had reduced empathic concern, increased SA, and a compressed macroscale organization of CT, indicating selective co-alterations in empathy and cortical structure. Future work should build on these novel insights in both the general and incarcerated populations to inform the treatment of psychopathic traits such as reduced empathy.

## Supplementary Material

Tables S6–S10

Key Resources Table

## Footnotes

Supplementary material cited in this article is available online at https://doi.org/10.1016/j.bpsgos.2026.100695.
